# Interference of Small RNAs in *Fusarium graminearum* through FgGMTV1 Infection

**DOI:** 10.3390/jof8121237

**Published:** 2022-11-22

**Authors:** Shuangchao Wang, Shaojian Ruan, Mingming Zhang, Jianhua Nie, Clement Nzabanita, Lihua Guo

**Affiliations:** 1 State Key Laboratory of Plant Diseases and Insect Pests, Institute of Plant Protection, Chinese Academy of Agricultural Sciences, 100193 Beijing, China; 2 Functional and Evolutionary Entomology, Gembloux Agro-Bio Tech, University of Liège, 5030 Gembloux, Belgium

**Keywords:** RNA silencing, FgGMTV1, virus-derived small RNA

## Abstract

Small RNA (sRNA) plays a central role in RNA silencing in fungi. The genome of Fusarium graminearum gemytripvirus 1 (FgGMTV1) is comprised of three DNA segments: DNA-A, DNA-B, and DNA-C. DNA-A and DNA-B are associated with fungal growth and virulence reduction. To elucidate the role of RNA silencing during the interactions of fungi and viruses, the sRNA profiles of *F. graminearum* in association with FgGMTV1 were established, using an FgGMTV1-free library (S-S), a library for infection with the DNA-A and DNA-B segments (S-AB), and a library for infection with the DNA-A, DNA-B, and DNA-C segments (S-ABC). A large amount of virus-derived sRNA (vsiRNA) was detected in the S-AB and S-ABC libraries, accounting for 9.9% and 13.8% of the total sRNA, respectively, indicating that FgGMTV1 triggers host RNA silencing. The total numbers of sRNA reads differed among the three libraries, suggesting that FgGMTV1 infection interferes with host RNA silencing. In addition, the relative proportions of the different sRNA lengths were altered in the S-AB and S-ABC libraries. The genome distribution patterns of the mapping of vsiRNA to DNA-A and DNA-B in the S-AB and S-ABC libraries were also different. These results suggest the influence of DNA-C on host RNA silencing. Transcripts targeted by vsiRNAs were enriched in pathways that included flavin adenine dinucleotide binding, protein folding, and filamentous growth.

## 1. Introduction

RNA silencing plays an important role in the gene regulation of fungi, plants, and animals [1,2]. Small regulatory RNAs are core molecules expressed during the RNA silencing process in eukaryotic organisms. Since the first discovery of small RNAs (sRNAs) in *Escherichia coli* in 1984 and the first discovery of small silencing RNAs in 1993, the control of gene expression through small non-coding RNAs has been highlighted as an important transcriptional regulatory mechanism [3,4,5]. Long double-stranded RNA (dsRNA) triggers the RNA silencing system and is cleaved by Dicer-like (DCL) proteins into short interfering RNAs (siRNAs). RNA-dependent RNA polymerase (RdRp) amplifies the siRNA population [6]. In eukaryotes, siRNAs induce the silencing of target genes at both the transcriptional and post-transcriptional levels. Here, they are defined by their short length (18–30 nucleotides) and association with the proteins of the Argonaute family (AGO), which can recognize target mRNAs and lead to their reduced expression [7].

In plants, 21 and 24 nucleotides (nt) are produced from DCL 4 and DCL 3 cleavage [8]. AGO has a preference for 5′-end nucleotides. For example, AGO 2 and AGO 4 preferentially recruit small RNAs with 5′-end adenosine (A), while AGO1 carries microRNAs (miRNAs) that favor 5′-end uridine (U) [9,10,11]. Although the *DCL* and *AGO* genes have been well studied in plants and animals, the characterization of the critical proteins involved in fungal RNA silencing is relatively poor. In fungi, RNA silencing was first discovered in *Neurospora crassa*. The DCL, AGO, and RdRp proteins are also three critical enzyme families for fungal sRNA production [12,13]. Different size classes of sRNAs are processed by different DCLs and AGOs. *C. parasitica* is the best-studied virus-infected fungus. The role of RNA silencing during fungus–virus defense and counter-defense was first reported in *Cryphonectria parasitica*. DCL 2, but not DCL 1, is responsible for 21 and 22 nt virus-derived small RNAs (vsiRNAs) derived from Cryphonectria parasitica hypovirus 1 (CHV-1) [14]. In addition, compared with DCL 1, DCL 2 has higher antiviral activity in several fungi [15,16].

Fungal viruses exist in various fungi, and they mostly cause asymptomatic infection. At present, the majority of the genome types of fungal viruses are RNA, including dsRNA and ssRNA, and a few DNA viruses have been reported. Sclerotinia-sclerotiorum-hypovirulence-associated DNA virus 1 (SsHADV-1) was the first fungal DNA virus, and it was isolated from *S. sclerotiorum*. Recently, the transcriptional response of *S. sclerotiorum* to the DNA virus SsHADV-1 was published [17]. It was revealed that the expression of antiviral RNA silencing components was suppressed, which indicated that RNA silencing played a critical antiviral role in the DNA-virus-infected fungi.

*Fusarium graminearum* is a major pathogen involved in Fusarium head blight (FHB) which threatens the production of small-grain cereals. In addition, many kinds of mycotoxins, such as deoxynivalenol, can be produced by this fungal species, which causes serious food safety concerns [18]. At present, there have been over ten mycoviruses discovered from *F. graminearum*. Among them, Fusarium graminearum gemytripvirus 1 (FgGMTV1) is a tripartite circular single-stranded DNA (ssDNA) mycovirus. The genome of FgGMTV1 has three circular ssDNA segments (DNA-A, DNA-B, and DNA-C) [19]. This virus belongs to a new family, *Genomoviridae*. In addition, *F. graminearum* shows different symptoms when infected with different viral DNA segments of FgGMTV1 [19].

Previously, we demonstrated that DNA-A and DNA-B were essential for viral replication and transmission. They are responsible for changes in fungal morphology and hypovirulence. DNA-C exists as satellite-like DNA. A fungus with DNA-C exhibits a normal morphology and pathogenicity. The roles of RNA silencing during FgGMTV1 infection and fungal counter-defense have not yet been explored. In this study, we analyzed sRNA profiling in FgGMTV1-infected *F. graminearum* and obtained the distribution of vsiRNA in different segments of the FgGMTV1 genome. The 5′-terminal nucleotide bias of vsiRNA, strand bias, and function of the host genes targeted by vsiRNA were also analyzed. We sought to understand how different segments of FgGMTV1 infection cause different phenotypes in *F. graminearum* from the point of view of fungal RNA silencing.

## 2. Materials and Methods

### 2.1. Collection of Fungal Isolates and Culture Conditions

The virus-free strain was the *F. graminearum* strain PH-1. Strain S-AB was obtained through the transformation of PH-1 with an infectious clone of the DNA-A and DNA-B segments of FgGMTV-1. Strain S-ABC was obtained through the transformation of PH-1 with an infectious clone of the DNA-A, DNA-B, and DNA-C segments of FgGMTV-1 [19]. All strains were kept on a potato dextrose agar (PDA) plate at 25 °C in darkness. Fresh mycelia were placed in the middle of a 9 cm PDA plate and cultured for 4 days for RNA isolation.

### 2.2. RNA Isolation and sRNA Deep Sequencing

Mycelial masses were collected at the same time point (4-day-long culture). Total RNA was extracted with an RNA extraction kit (Tiangen, Beijing, China). The quality was determined by HPLC. Additionally, the qualified RNA was used for high throughput sequencing using a DNBSEQ-T7 system (MGI Tech, Shanghai, China). The original data obtained by sequencing were named raw reads. By mapping the sequencing data with the reference genome sequence, contamination or confusion in the sample were judged. Before data analysis, low-quality data and adapters were removed. Samples with high genome mapping rate were used for the following analysis. The sRNA sequencing raw data were submitted to the National Center for Biotechnology Information website (https://www.ncbi.nlm.nih.gov/, accessed on 25 October 2022) under project No. PRJNA884307.

### 2.3. High-Throughput Sequencing Data Analysis

Clean and qualified sRNAs were selected for the following analysis. Three databases were used for non-coding RNA identification. miRBase V22 (http://www.mirbase.org/, accessed on 25 October 2022) contains a large amount of information on miRNAs and target genes of animals and plants. RNAcentral V16.0 (https://rnacentral.org/, accessed on 25 October 2022) is a non-coding RNA database, and it provides the most comprehensive and up-to-date information on non-coding RNA of different species. The Rfam V13.0 database (https://rfam.xfam.org/, accessed on 25 October 2022) is a collection of RNA families, each represented by multiple sequence alignments, consensus secondary structures and covariance models. Based on the taxonomy ID of the species, we downloaded the non-coding RNA sequence of this species from miRBase and RNAcentral, and then mapped reads to these sequences to identify known non-coding RNAs. After these, the un-annotated reads were mapped to Rfam. However, if there was no non-coding RNA in miRBase and RNAcentral, reads were mapped to Rfam directly. Based on the AASRA theory (http://biorxiv.org/content/early/2017/05/01/132928, accessed on 25 October 2022), we chose the best mapping of each read, and then calculated the tags per million reads (TPM) expression of each non-coding RNA. After obtaining the expression of all non-coding RNAs, principal component and correlation analyses were carried out. According to the results of the correlation analysis and principal component analysis, the similarity between samples can be judged. The better the repeatability, the greater the correlation coefficient, and the closer the cluster in the principal component analysis diagram.

### 2.4. VsiRNA Identification and Their Function Analysis

All sRNAs were mapped to genome segments (DNA-A, DNA-B and DNA-C) of FgGMTV1. Targets of vsiRNA were predicted using TargetScan (http://www.targetscan.org/cgibin/targetscan/data_download.cgi?db=vert_61, accessed on 25 October 2022). All targets were clustered into GO terms in the database (http://www.geneontology.org/, accessed on 25 October 2022). To identify target-enriched pathways, Encyclopedia of Genes and Genomes (KEGG, http://www.genome.jp/kegg) pathway analysis was performed. The corrected *p*-value ≤ 0.05 was used for both GO and KEGG significant calculations. Differences were compared among three groups (S-AB vs. S-S, S-ABC vs. S-S, and S-AB vs. S-ABC).

## 3. Results

### 3.1. Small RNAs Are Differentially Expressed in FgGMTV1-Infected and -Free Fusarium Graminearum

To examine whether sRNA accumulation is affected by FgGMTV1 infection, we prepared three isogenic *F. graminearum* strains as sequencing samples: S-S (virus-free strain), S-AB (S-S strain infected with DNA-A and DNA-B segments of FgGMTV1), and S-ABC (S-S strain infected with DNA-A, DNA-B and DNA-C segments of FgGMTV1). Deep sequencing obtained averages of 34,891,470, 20,999,020 and 37,654,931 raw reads from three groups of data for the FgGMTV1-infected (S-AB, S-ABC) and FgGMTV1-free (S-S) libraries, respectively. After removing the adaptors and filtering out low-quality tags and contaminants due to adaptor ligation, averages of 34,773,553, 20,712,141 and 37,559,916 clean reads were obtained from the three groups for the FgGMTV1-infected (S-AB, S-ABC) and FgGMTV1-free (S-S) libraries, respectively. All clean reads ratios were greater than 95%, indicating that quality control was sufficient and the data were reliable. The sRNA quantity of S-ABC is significantly less than that of S-AB and S-S. Normally, viral infection triggers RNA silencing and induces sRNA production. The lower sRNA accumulation in the S-ABC library suggests that DNA-C may encode an RNA silencing suppressor that inhibits host RNA silencing.

**Figure 1:**
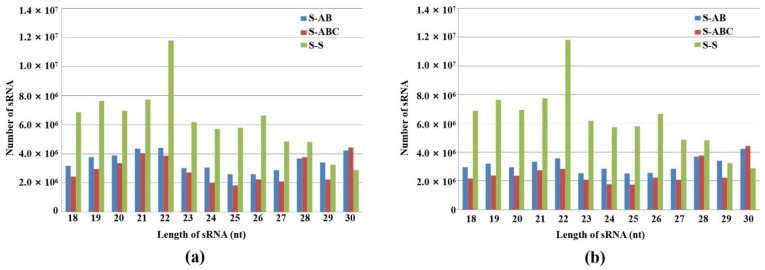
Size distribution and abundance of sRNAs in FgHV1-infected *F. graminearum*. (**a**) 18–30 nt total sRNA distribution in S-AB, S-ABC and S-S. (**b**) 18–30 nt total sRNA distribution in S-AB, S-ABC and S-S after filtering out FgGMTV1-derived sRNA. The *y*-axis indicates the reads number of sRNAs, and the *x*-axis represents the length distribution. S-S (virus-free strain), S-AB (S-S strain infected with DNA-A and DNA-B segments of FgGMTV1), S-ABC (S-S strain infected with DNA-A, DNA-B and DNA-C segments of FgGMTV1).

Furthermore, we analyzed the size distribution of sRNA in the three strain groups. As shown in Figure 1a, the numbers of sRNA with lengths between 18 and 30 nt in virus-infected strains (S-AB, S-ABC) were all significantly reduced compared to the virus-free strain (S-S). In the virus-free (S-S) and S-AB libraries, the 22-nt sRNAs were the most dominant class, accounting for 14.5% and 9.8% of the sRNAs in the FgGMTV1-free (S-S) and FgGMTV1-infected (S-AB) libraries, respectively. However, 30-nt sRNAs were the most dominant class, accounting for 11.7% of the sRNAs in the FgGMTV1-infected (S-ABC) library. This result is different from the sRNA distribution pattern of some plant species [20,21,22]. After filtering out FgGMTV1-derived sRNA, FgGMTV1-infected *F. graminearum* hosts (S-AB, S-ABC) produced less sRNA than the virus-free strain (S-S) (Figure 1b). There was also no significant effect on the number of 29-nt sRNA reads in the FgGMTV1-infected library (S-AB) compared to the FgGMTV1-free library (S-S) (Figure 1b). Moreover, the amount of 30-nt sRNA in libraries infected with FgGMTV1 (S-AB and S-ABC) was significantly increased compared to libraries not infected with FgGMTV1. The overall size distribution pattern of sRNAs was significantly different between virus-infected (S-AB, S-ABC) and virus-free libraries (S-S), suggesting that infection with different segments of FgGMTV1 could affect the function of DCL, RdRp and other key compounds responsible for sRNA accumulation in host *F. graminearum*.

### 3.2. Polarity and Size Profiling of FgGMTV1-Derived siRNA in F. graminearum Infected by Different Segments of FgGMTV1

To determine viral RNA-triggered RNA silencing and FgGMTV1-derived sRNA (vsiRNA) size properties in the host, sRNAs of 18–30 nt in the libraries were retrieved and filtered for matching with different segments of the FgGMTV1 genome. The redundant vsiRNA reads were 4,502,173 (S-AB) and 5,245,399 (S-ABC), which accounted for 9.9% and 13.8% of the total sRNA reads, respectively. The large amount of vsiRNA production in FgGMTV1-infected *F. graminearum* suggests that FgGMTV1 induces RNA silencing of the host to defend against the virus. In addition, we found that DNA-B produced significantly more vsiRNA in S-AB than in S-ABC. Compared with S-ABC, the 18–30 nt vsiRNA mapped to the DNA-A genome only increased by 9.6%, while the mapping to the DNA-B genome increased by 137.6% in S-AB, indicating that the FgGMTV1 segment DNA-C inhibited the silencing of DNA-B, and that DNA-B becomes a hot target for host RNA silencing when DNA-C is absent. The polarity of vsiRNA analysis revealed that more vsiRNAs originated from the sense strand (+) than the antisense strand (−) of the viral genome. 

**Figure 2:**
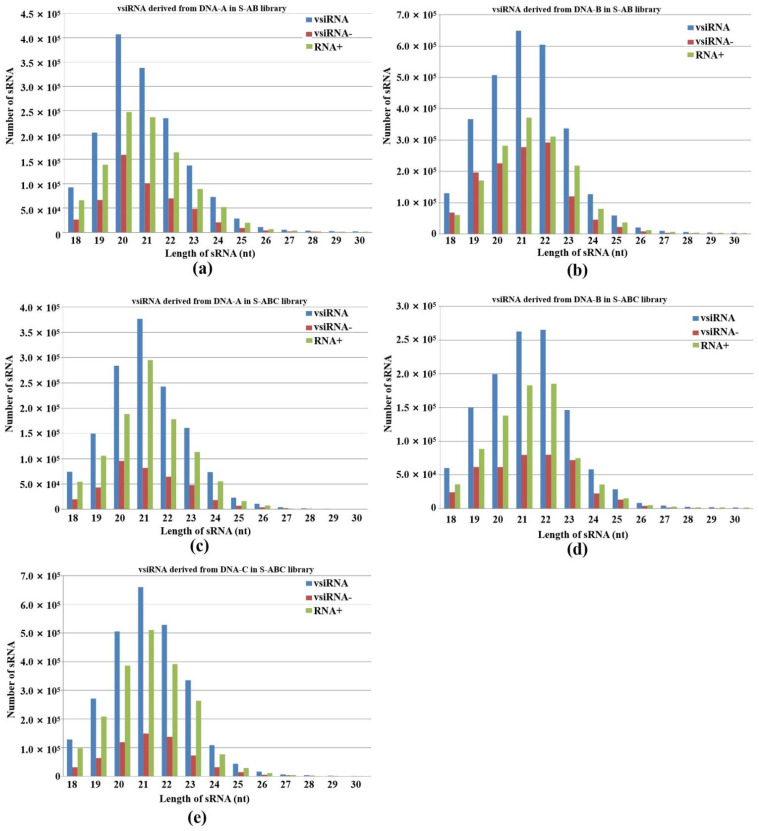
Abundance and size distribution of vsiRNAs along the different genome segments of FgGMTV1. vsiRNAs originating from the positive strand (vsiRNA+) and the negative strand (vsiRNA-) of (**a**) DNA-A of S-AB, (**b**) DNA-B of S-AB, (**c**) DNA-A of S-ABC, (**d**) DNA-B of S-ABC, (**e**) DNA-C of S-ABC were clustered based on size, ranging from 18 to 30 nt. The *y*-axis indicates the numbers of sRNAs and the *x*-axis represents the length distribution. Total vsiRNA is represented by blue bars, vsiRNA- by red bars, and vsiRNA+ by green bars. S-S (virus-free strain), S-AB (S-S strain infected with DNA-A and DNA-B segments of FgGMTV1), S-ABC (S-S strain infected with DNA-A, DNA-B and DNA-C segments of FgGMTV1).

Our results showed that 20-, 21- and 22-nt vsiRNA reads dominated the vsiRNA sequences examined (Figure 2). This shows a certain similarity to plant virus-derived siRNA profiles: in plants, 21-nt virus-derived RNA, which is mainly produced from DCL4 cleavage, is also present [23,24,25]. We also found that the number of 20-nt and 21-nt vsiRNAs from DNA-A accounted for the largest proportion in S-AB and S-ABC, respectively (Figure 2a,c). In addition, 21-nt vsiRNA in S-AB and 22-nt vsiRNA in S-ABC from DNA-B showed peaks (Figure 2b,d). In plants, the 22-nt siRNAs mediate translational repression and stress adaption [22]. Thus, the 22-nt siRNAs from DNA-B of FgGMTV1 may repress the gene translation of *F. graminearum* and confer hypovirulence after viral infection. As shown in Figure 2e, the 21-nt vsiRNA from DNA-C peaked in S-ABC. These results showed that vsiRNA size distributions of DNA-A, B, and C are different, indicating that the two FgDCLs are attracted to different segments of FgGMTV1. 

### 3.3. Distribution of vsiRNA along the Different Genome Segments of FgGMTV1 Genome

To examine the genomic distribution of vsiRNAs, the 5′-terminus of vsiRNA from FgGMTV1-infected groups (S-AB, S-ABC) was mapped to the corresponding genome segment of FgGMTV1 according to their polarities and genomic locations. When mapped along the DNA-A, DNA-B and DNA-C genomes in S-AB and S-ABC, the amount of vsiRNA from the positive strand was higher than from the negative strand. The amount of vsiRNA in the DNA-A and DNA-B genomes of S-ABC was lower than in S-AB. 

**Figure 3:**
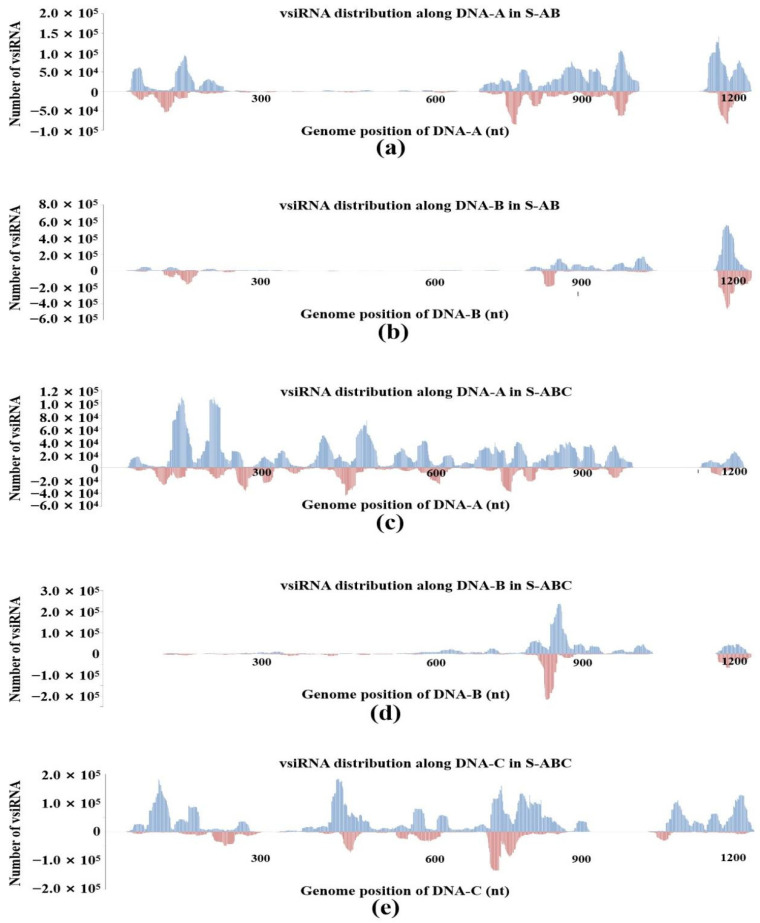
VsiRNA genome mapping on different segments of FgGMTV1 in S-AB and S-ABC. (**a**) vsiRNA from S-AB mapping along DNA-A. (**b**) vsiRNA from S-AB mapping along DNA-B. (**c**) vsiRNA from S-ABC mapping along DNA-A. (**d**) vsiRNA from S-ABC mapping along DNA-B. (**e**) vsiRNA from S-ABC mapping along DNA-C. The abundance of reads matching the positive strand is represented by the blue bar, whereas the abundance of reads mapping to the negative strand is represented by the red bars. Genomic coordinates are shown on the x-axis and the point cumulative abundance of all reads is shown on the y axis. S-S (virus-free strain), S-AB (S-S strain infected with DNA-A and DNA-B segments of FgGMTV1), S-ABC (S-S strain infected with DNA-A, DNA-B and DNA-C segments of FgGMTV1).

The distribution patterns of vsiRNA in the same segment of S-AB and S-ABC were different. In S-AB, vsiRNA is non-randomly distributed along the DNA-A genome. The hotspots of vsiRNA mapped to the DNA-A genome mainly appeared in 5′, 3′-UTR and the ORF region, and there were few vsiRNA germination sites in the middle of the DNA-A genome (Figure 3a). In S-ABC, vsiRNAs nearly saturated the viral DNA-A genome, both in coding and noncoding regions, although some spiking hotspots could be detected (Figure 3c). In the S-AB and S-ABC groups, vsiRNAs were distributed along the DNA-B genome similarly in a non-random pattern, with multiple peaks in the 5′- and 3′-UTR of the DNA-B genome. Notably, S-AB has a higher vsiRNA accumulation hotspot in the 3′-terminal region of DNA-B than S-ABC (Figure 3b,d). In S-ABC, vsiRNA is distributed along the coding and noncoding regions of DNA-C, nearly saturating the viral genome (Figure 3e). In addition, we constructed 21-nt and 24-nt vsiRNAs profiles matching on different segments of FgGMTV1. In S-ABC, there are more 21-nt vsiRNAs from DNA-A and DNA-B than 24-nt vsiRNAs, and more 24-nt vsiRNAs from DNA-C than 21-nt vsiRNA, indicating that DNA-A, DNA-B and DNA-C may be cleaved by different DCLs.

### 3.4. 5′-Terminal Nucleotides of the FgGMTV1-Derived siRNAs Showed a Preference for U Residue

**Figure 4:**
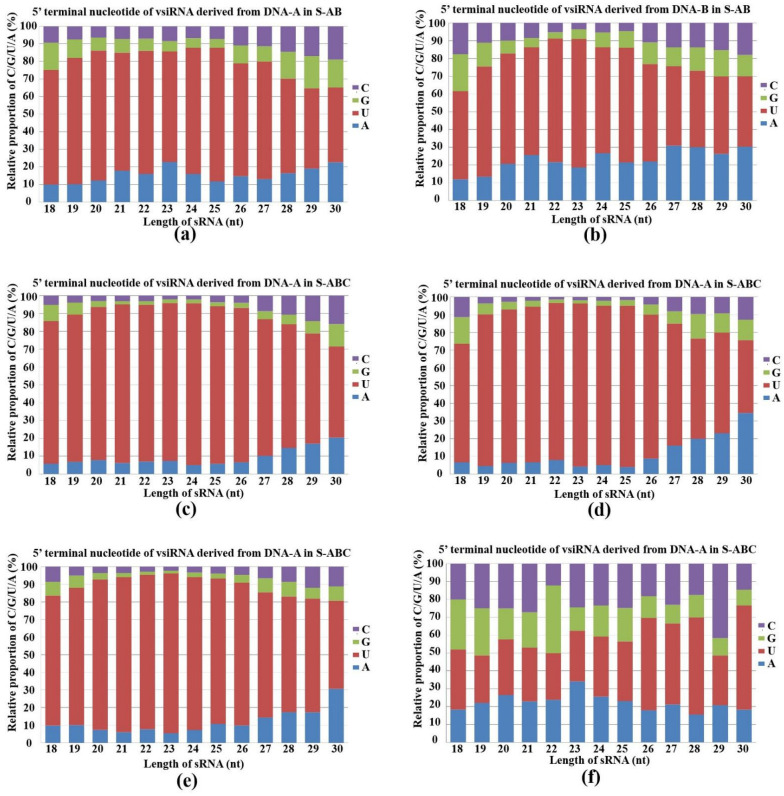
Composition of the 5′-terminal nucleotide among vsiRNAs. Composition of the 5′-terminal nucleotide of 18–30-nt siRNA derived from (**a**) DNA-A in S-AB, (**b**) DNA-B in S-AB, (**c**) DNA-A in S-ABC, (**d**) DNA-B in S-ABC, (**e**) DNA-C in S-ABC, (**f**) *F. graminearum* genome in S-S. The x-axis represents vsiRNA length, ranging from 18- to 30-nt and the y-axis shows the composition of G/C/A/U at 5′-terminal nucleotides. S-S (virus-free strain), S-AB (S-S strain infected with DNA-A and DNA-B segments of FgGMTV1), S-ABC (S-S strain infected with DNA-A, DNA-B and DNA-C segments of FgGMTV1).

AGOs load sRNA with different preferences for their 5′-terminal nucleotides in plants and animals [26]. *Arabidopsis* AGO 2 and AGO 4 mainly bind sRNAs beginning with 5′-terminal adenosine (A), whereas AGO 1 harbors microRNAs (miRNAs) that favor 5′-terminal uridine (U) [27]. To determine the potential interactions between FgGMTV1-derived siRNAs and distinct *F. graminearum* AGO complexes, we analyzed the compositions of 5′-terminal nucleotides of vsiRNAs. The U residue was the most abundant nucleotide in the 5′-terminal position of FgGMTV1-derived siRNAs in S-AB and S-ABC. Specifically, the 18–30 nt siRNAs derived from DNA-A, DNA-B and DNA-C in S-AB and S-ABC all showed a preference for the 5′-terminal U (Figure 4a–e). However, the proportion of 5′-terminal U of DNA-A- and DNA-B-derived siRNAs in S-ABC was higher than that in S-AB, respectively. We also found that sRNA germinated from *F. graminearum* hosts showed no significant preference for U at the 5′-terminal position (Figure 4f). For example, the 22-nt sRNA of *F. graminearum* showed a preference for guanine, whereas the 23-nt sRNA showed a preference for A in S-S (virus-free strain). These results suggest that multiple AGOs play important roles in *F. graminearum* sRNA-mediated target cleavage and that certain AGOs may be activated as major loading components of vsiRNA in the RISC complex.

### 3.5. Host Genes Targeted by vsiRNAs Have Variable Functions

**Figure 5:**
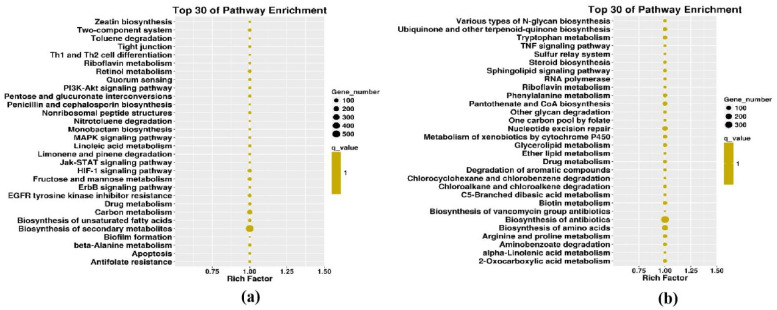
KEGG pathway enrichment from DNA-A- and DNA-B-derived sRNA in S-AB. (**a**) DNA-A-derived sRNA target-enriched KEGG pathways in S-AB. (**b**) DNA-B-derived sRNA target-enriched KEGG pathways in S-AB.

To identify specific vsiRNA functions, we analyzed the targets of vsiRNA. GO enrichment and KEGG pathway analysis were performed. Because DNA-A and -B genome segments of FgGMTV1 infection result in abnormal fungal colony morphology, it is of great interest to analyze the KEGG pathway enriched in the S-AB library (Figure 5). Specific GO terms for DNA-A-derived siRNA targets in S-AB were enriched in cellular components such as the nucleus, intracellular, and preribosome large subunit precursors. Unlike S-AB, the targets of DNA-A-derived siRNA in S-ABC were mainly related to flavin adenine dinucleotide binding, protein folding, and filamentous growth GO terms. The most common targets of DNA-B-derived siRNAs in S-AB included heme binding, filamentous growth, and conidiophore development, while their targets were cytoplasm- and intracellular-related cellular components in S-ABC. For DNA-C-derived siRNA targets in S-ABC, nucleotide binding was the most significant enriched GO term. We also constructed enriched KEGG pathways for each component-derived siRNA target. Their targets were involved in a variety of pathways, including Jak−STAT, MAPK signaling, ubiquinone and other terpenoid−quinone biosynthesis, nucleotide excision repair, and tyrosine metabolism. Notably, 121 targeted genes of DNA-C-derived siRNAs from S-ABC were involved in tyrosine metabolism.

## 4. Discussion

RNA silencing is one of the most important pathways for virus defense. Fungi, plants and animals have similar RNA silencing components and process [28]. At the first stage, virus infection triggers host RNA silencing. Long double strand RNA are cleaved into siRNA for RISC loading and target RNA cleavage. Thus, sRNA is the critical molecule for RNA silencing [29,30]. There is a relative lack of research on fungal viruses compared to plant and animal viruses. However, with the application of new methods, such as high-throughput sequencing, fungal viruses are being discovered at a quicker rate [31,32,33]. In addition, deep sequencing of RNA could also be used in fungi–virus defense and counter-defense research. The profiles of virus-derived sRNAs from fungal hosts such as *Botrytis* suggest that fungal viruses trigger similar RNA silencing pathways in fungi as in plants and animals [34,35]. As a counter-defense, viruses including fungal viruses suppress host RNA silencing via different methods [36,37]. In this study, we characterized the sRNA profiles of *F. graminearum* infected with different genome segments of an ssDNA mycovirus, FgGMTV1. Nearly ten percent of the 18–30-nt total sRNA reads in *F. graminearum* were derived from the virus. The high-resolution sRNA maps of different virus genome segments were compared. In the presence of DNA-C segment, sRNA profiles and vsiRNA characteristics were greatly altered.

Our results showed that there were more sRNAs accumulated in S-AB than in S-ABC, which indicates that DNA-C probably encodes an RNA silencing suppressor (RSS). Until now, there have been four RSSs identified from mycoviruses, including CHV1-encoded p29, RnMyRV3-encoded s10, FgV1-encoded ORF 2 and CHV4-encoded p27 [38,39,40,41]. We analyzed the ORFs on DNA-C and compared the sequences with identified RSSs encoded by fungi, plant, and animal viruses. The p18 or other predicted ORFs might be potential new RSSs and further experiments can be designed for RNA silencing suppression.

**Figure 6:**
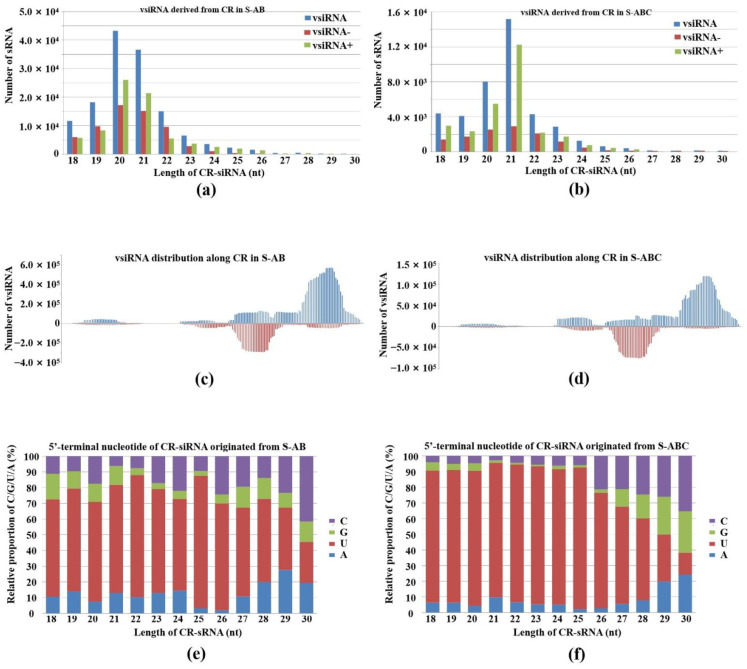
Characterization of vsiRNA derived from the CR sequence of FgGMTV1. (**a,b**) Size distribution, (**c,d**) genome mapping and (**e,f**) 5′-terminal nucleotide of CR-siRNAs originating from S-AB and S-ABC libraries, respectively. S-AB (S-S strain infected with DNA-A and DNA-B segments of FgGMTV1), S-ABC (S-S strain infected with DNA-A, DNA-B and DNA-C segments of FgGMTV1).

The common region (CR) is critical for circular DNA virus replication and existence, including the origin for rolling circle. The CR of FgGMTV1 is nearly 200 nt. Normally, CR exists as a steam loop structure, which is the best position to generate sRNA. We detected CR-derived vsiRNA accumulation in S-AB and S-ABC and analyzed the CR-derived siRNA size distribution and genome mapping in detail (Figure 6). CR-derived siRNA was higher in S-AB than S-ABC. The vsiRNA reads number from the CR region in S-ABC was reduced by 77.8% compared to S-AB. The 20- and 21-nt showed the highest peaks for CR-siRNAs in S-AB and S-ABC, respectively (Figure 6a,b). The CR-siRNA distribution along the genome is shown in Figure 6c,d, which has similar hotspots and coldspots as in S-AB and S-ABC. In addition, the 5′ terminal nucleotide of most CR-siRNAs is U in both S-AB and S-ABC (Figure 6e,f). These results suggest that CR, with a complex secondary structure, is also an important target of host RNA silencing defense systems. We also analyzed the pathways targeted by vsiRNA derived from CR. The pectin catabolic process and cellular protein localization terms, RNA transport, and cysteine/methionine metabolism pathways were enriched by CR-derived siRNA-targets of S-AB and S-ABC. Host antiviral silencing systems of fungi may be repressed by DNA-C in S-ABC.

In animals and plants, different DCLs are responsible for different sized sRNA cleavages. There are four DCLs (DCL 1, 2, 3 and 4) in *F. graminearum* [42], but their cleavage sRNA sizes remain unexplored. The 22-nt sRNA was the peak for virus-free *F. graminearum*. However, the 30-nt class of sRNAs, followed by the 21-nt class, accounted for the highest proportion in the FgGMTV1-infected library, S-ABC. Unlike the S-ABC library, the 22- and 21-nt classes of sRNAs held the primary part of the FgGMTV1-infected library, S-AB. We also found that the highest peak for vsiRNA from each FgGMTV1 segment was 21 nt, except for DNA-A-derived siRNA from S-AB (peak at 20 nt) and B-derived siRNA from S-ABC (peak at 22 nt). These results suggest that FgGMTV1 infection influences the cleavage efficiency of different DCLs. In addition, the preferred 5′-terminal nucleotide of each size of vsiRNA was U. This indicated that one of two AGOs plays the main role in *F. graminearum* upon FgGMTV1 infection. As we know, AGO 1 preferentially binds sRNAs beginning with U in *Arabidopsis*. Based on sequence alignment, we can further explore the functional AGOs of *F. graminearum* that have the highest identities of AGO 1 encoded by *Arabidopsis*. Combined with vsiRNA genome mapping and target prediction, our results will guide further research into critical components during defense and counter-defense between *F. graminearum* and FgGMTV1.

## Data Availability

The sequencing data from this study have been submitted (http://www.ncbi.nlm.nih.gov/sra) to the NCBI Sequence Read Archive (SRA) under accession no. BioProject ID: PRJNA884307.
